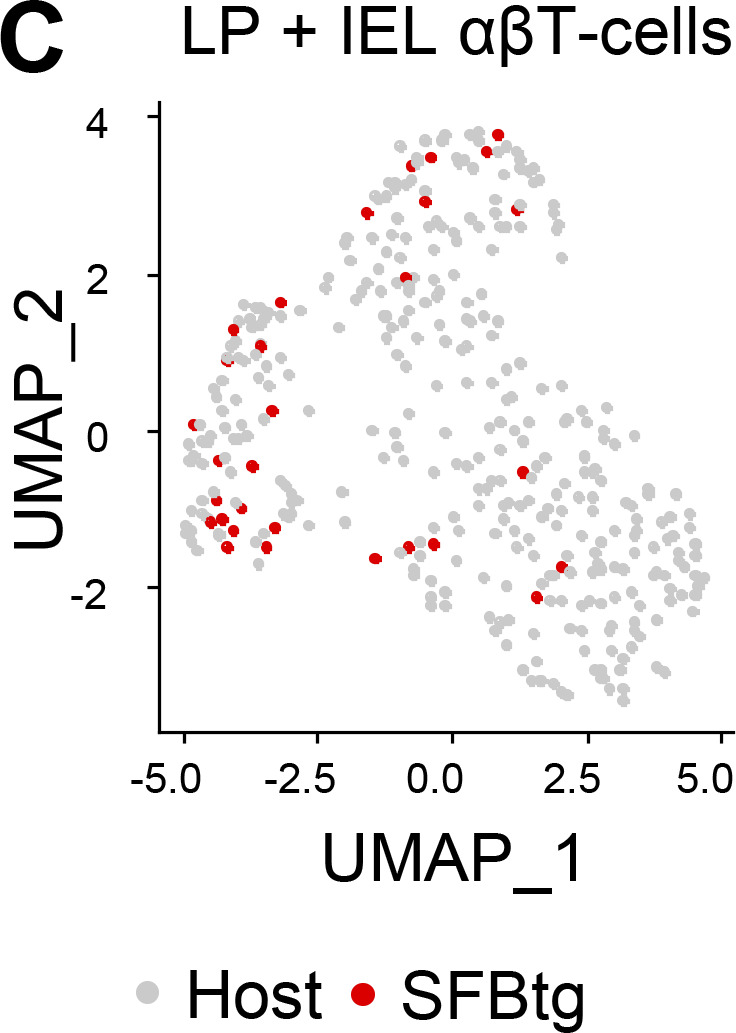# Correction: Segmented filamentous bacteria–induced epithelial MHCII regulates cognate CD4^+^ IELs and epithelial turnover

**DOI:** 10.1084/jem.2023019411132023c

**Published:** 2023-11-21

**Authors:** Tomáš Brabec, Martin Schwarzer, Katarína Kováčová, Martina Dobešová, Dagmar Schierová, Jiří Březina, Iva Pacáková, Dagmar Šrůtková, Osher Ben-Nun, Yael Goldfarb, Iva Šplíchalová, Michal Kolář, Jakub Abramson, Dominik Filipp, Jan Dobeš

Vol. 221, No. 1 | https://doi.org/10.1084/jem.20230194 | October 30, 2023

The authors regret that in the originally published Fig. 8, the dots in panel C were not labeled correctly. The panel appears here with the corrected “Host” and “SFBtg” labels. This correction does not change the original conclusions of the article, and the figure legend remains unchanged. The error appears in PDFs downloaded before November 10, 2023.

**Figure 8:**